# A Simple and Practical Method of Sterilizing Soft-Rubber Catheters

**Published:** 1897-06

**Authors:** James H. Montgomery

**Affiliations:** Erie, Pa.; 115 West 8th Street


### A SIMPLE AND PRACTICAL METHOD OF STERILIZING SOFT-RUBBER CATHETERS.

BY JAMES H. MONTGOMERY, A. B., M. D., ERIE, PA.

For the past four years I have sterilized soft-rubber catheters in a simple manner, one within the reach of every physician, and have had such good results from the method, both clinically and bacteriologically, that the method may be of some interest to those not acquainted with any method of rendering catheters free from danger of infections. This method is referred to by Park in his "System of American Surgery," Vol. 1, p. 374, where a brief outline of the method is given. So far as I know, it has never been published, and is entirely original.

The principle of the process is exactly the same as that employed to sterilize culture media in test-tubes, the catheter taking the place of the media, and the container the test-tube. The containers are glass gauge-tubes, such as are used on steam boilers to denote the water-level. These are sold in various sizes and lengths at most machine-shops. The most useful size is the 16 x $\frac{5}{8}$ inches (external measurement). They can be purchased by the dozen at a cost of about ten or twelve cents apiece. These tubes are fully cleansed by washing, dried, the ends plugged with cotton, and then sterilized by the heat of an ordinary kitchen range. In practice I find a temperature of about 350° F. can be reached before the cotton scorches. The tubes should be subjected twice to this process. They are then in a condition to receive the catheter, which is sterilized as follows: The catheter is boiled in any convenient utensil for thirty minutes, then dried with a piece of gauze (previously sterilized) and dropped in the prepared tube, the ends of which, containing the cotton plugs, are covered with a bit of rubber plaster, to keep the cotton in place.

Those possessing a steam sterilizer can sterilize the catheters by steam for thirty minutes, as dressings are sterilized, and then put them directiy into the tube. The steam sterilizing obviates the necessity of wiping the catheters, as must be done when they are boiled, and thus eliminates a source of possible infection. If an apparatus that permits sterilizing by steam under pressure can be used, the catheters can be placed in the sterilized tube and then sterilized, thus avoiding any handling after they have been rendered aseptic.

To use the catheter thus sterilized, it is dropped on a clean towel, or sterilized gauze, the hands disinfected, the catheter lubricated with sterilized glycerin, or boroglycerid 1-16, and then introduced in the ordinary manner. The employment of glycerin as a lubricant is important, as the inside of the catheter can be thoroughly cleansed under a stream of running water, an impossibility if any vaselin or oily preparation is employed for lubricating purposes.

Catheters sterilized by this method have given the best of results clinically, especially in cases of purulent urine from enlargement of the prostrate gland, and in irrigation of the deep urethra. Attempts to obtain a culture of any sort from a catheter disinfected in this manner have always been negative. I think this test shows that the catheter is an aseptic condition, certainly far more so than can be obtained by any method now used. It is fair to assert that if culture media can be thoroughly sterilized in a test-tube, a catheter can also be rendered sterile by employing this method. I find that a soft-rubber catheter can be boiled repeatedly without injury, so that anyone can always sterilize a catheter before using, no matter under what conditions.

Practically it is simpler as well as quicker to sterilize a number of catheters at one time, and they can then be kept indefinitely and always ready for use. The objection that boiling or steaming the soft-rubber catheters is apt to destroy them is groundless, for I have subjected catheters to this process at least thirty times, and they are as good as ever. Even if the soft-rubber were injured after being submitted to this process a few times, what is the trivial cost of a catheter compared to the fact of having an instrument that can be passed into the bladder without danger of infecting the latter? When one considers the vast number of patients that have lost their lives from dirty catheters no argument is required to prove that only a sterilized instrument, no matter of what material constructed, should ever be passed into the bladder. Boiling water can always be obtained, and the catheter can soon be thoroughly disinfected and rendered safe to use.

*115 West 8th Street.*

